# Antithrombin as Therapeutic Intervention against Sepsis-Induced Coagulopathy and Disseminated Intravascular Coagulation: Lessons Learned from COVID-19-Associated Coagulopathy

**DOI:** 10.3390/ijms232012474

**Published:** 2022-10-18

**Authors:** Christian J. Wiedermann

**Affiliations:** 1 Institute of General Practice, Claudiana—College of Health Professions, 39100 Bolzano, Italy; christian.wiedermann@am-mg.claudiana.bz.it; 2 Department of Public Health, Medical Decision Making and HTA, University of Health Sciences, Medical Informatics and Technology—Tyrol, 6060 Hall in Tyrol, Austria

**Keywords:** antithrombin, biomarker, coagulopathy, COVID-19, disseminated intravascular coagulation, intervention, scoring, sepsis

## Abstract

Recent research has contributed significantly to our understanding of the pathogenesis of acute disseminated intravascular coagulation. COVID-19 can be considered as a new underlying condition of disseminated intravascular coagulation. In this narrative review, current evidence is presented regarding biomarker differences between sepsis-induced and COVID-19-associated coagulopathies, supporting the importance of acquired antithrombin deficiency in the early differential diagnosis of septic coagulopathy and its potential impact on treatment with endogenous anticoagulants. Establishing new scoring systems for septic coagulopathy in combination with endogenous anticoagulant biomarker activities may allow for the identification of those in the heterogeneous population of sepsis patients who are more likely to benefit from targeted specific treatment interventions.

## 1. Introduction

Life-threatening sepsis leading to organ dysfunction and death, which occurs as a result of the body’s dysregulated response to infection, remains a challenge for both physicians and clinical researchers. Endogenous anticoagulants protein C, thrombomodulin, tissue factor pathway inhibitor, and antithrombin (AT) have been intensively studied clinically as treatment options for excessive immune-thrombotic response, because coagulation and inflammation regulate each other through many mechanisms [1,2,3,4,5,6]. However, attempts to successfully treat sepsis with inflammation-modulating interventions, including endogenous anticoagulants, have failed, and a specific therapeutic agent for sepsis is currently lacking [7].

Acute coagulopathy is associated with reduced survival in patients with sepsis or coronavirus disease 2019 (COVID-19) and ranges from slight thrombocytopenia and derangements of coagulation parameters to fatal disorders such as disseminated intravascular coagulation (DIC). Systemic coagulation activation resulting from inflammatory events, such as infection or trauma, characterizes DIC [8]. The cascade of biomechanical events in DIC includes triggering of tissue factor-dependent coagulation by inflammatory mediators, inadequate anticoagulation by endogenous anticoagulants, and suppression of plasminogen activator inhibitor-1-mediated fibrinolysis. These pathophysiological changes are associated with endothelial dysfunction and microvascular clot formation, which, along with triggered organ dysfunction, are responsible for the worsened prognosis of affected patients.

Physiologic anticoagulation systems, including the AT–heparan sulfate system and thrombomodulin–protein C system, prevent unfavorable thrombosis; however, these systems are readily impaired during sepsis. For the successful development of a sepsis drug, it would have been necessary to select for intervention from the very heterogeneous group of sepsis patients who were similar in their response abnormalities [7]. In an observational study of genomic transcription, patients with sepsis were assigned to three different groups, which, in addition to a so-called adaptive and an inflammopathic phenotype, were also assigned to a coagulopathic group [9]. These three reaction types differed in terms of disease severity and outcome and could possibly respond differently to drug interventions.

This narrative review compares the coagulopathies of sepsis and COVID-19, with particular reference to the role of AT as a potential therapeutic intervention against DIC.

## 2. Diagnosis and Management of Sepsis-Induced Coagulopathy

The definition of DIC reported by the International Society on Thrombosis and Haemostasis (ISTH) in 2001 is “DIC is an acquired syndrome characterized by the intravascular activation of coagulation with loss of localization arising from different causes. It can originate from and cause damage to the microvasculature, which, if sufficiently severe, can produce organ dysfunction” [10]. This consensus designation assumed systemic activation of coagulation rather than consumption coagulopathy. The early stages of compensated systemic coagulation activation still were not adequately captured, neither by the Japanese Ministry of Health and Welfare (JMHW) scoring system from 1983 nor the ISTH score for overt DIC, which led to the development of the Japanese Association of Acute Medicine (JAAM) and the sepsis-induced coagulopathy (SIC) scoring systems. Because the JAAM score, which was widely used in Japan, was hardly used internationally, the Scientific Standardization Committee (SSC) of the ISTH released a new category of early-phase DIC arising from sepsis as the SIC in 2019 [11,12]. JAAM or SIC scoring are both able to cover the thrombosis part of DIC characteristic of sepsis but not the early part of bleeding in DIC from trauma or obstetric emergencies [13].

Activation of coagulation triggered by inflammation in sepsis leads to thrombosis, DIC, and eventually consumption coagulopathy, whereas coagulation activation in COVID-19 is mostly limited to thrombosis [14,15]. Increased plasma D-dimer levels, prolongation of prothrombin time (PT) and activated partial thromboplastin time (APTT), thrombocytopenia, and microangiopathic thrombosis occurring in multiple organs characterize DIC [12].

In addition, in patients with COVID-19, increased plasma D-dimer levels associated with the severity and complications of thromboses have been reported in many studies [16]. However, in COVID-19-associated coagulopathy, elevated activities of tissue factor and plasminogen activator inhibitor 1 (PAI-1) limit the degradation of cross-linked fibrin and the associated release of D-dimer. Levels of D-dimer compared with SIC are relatively lower in COVID-19-associated coagulopathy, which exhibits a marked increase in soluble tissue factor, but fewer alterations in platelets, antithrombin, and fibrinogen consumption, and less fibrinolysis [14].

**Figure 1:**
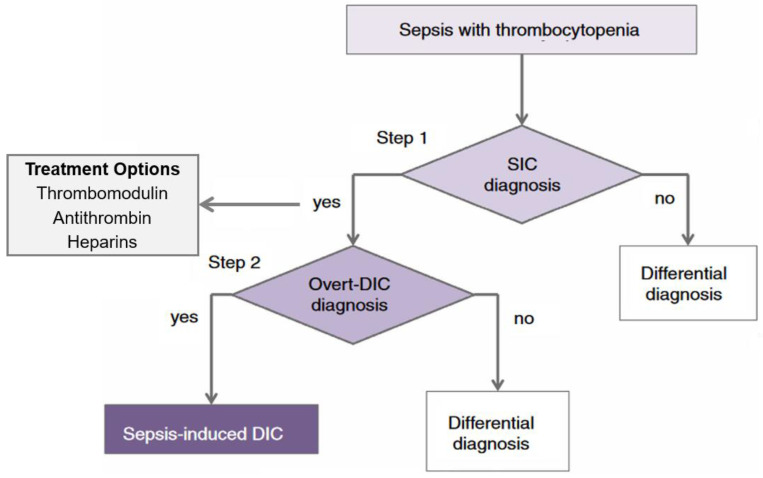
Two-step diagnosis for sepsis-associated DIC. The figure depicts an algorithm to diagnose sepsis-induced coagulopathy (SIC) and overt disseminated intravascular coagulation (DIC). Sepsis patients with thrombocytopenia (platelet count < 150 × 10^9^ × L^−1^) are screened by using SIC diagnostic criteria (Step 1), and then by using overt DIC diagnostic criteria (Step 2). The rationale for this approach is that SIC and overt DIC represent a continuum wherein the onset of SIC typically precedes that of overt DIC, and where early therapeutic intervention with anticoagulant therapy is most likely to be beneficial. Adapted with permission from Ref. [12]. Copyright ©2019, Blackwell Publishing.

The treatment recommendations for anticoagulant therapy in sepsis-associated DIC are controversial, and the anti-DIC interventions discussed are used quite differently internationally. A two-step diagnostic approach (Figure 1) has recently been recommended for sepsis patients with suspected SIC and sepsis-associated DIC before anticoagulant therapy [17].

### 2.1. Scoring of Sepsis-Induced Coagulopathy and DIC

Clinical trials of sepsis therapy have focused on intervening early in the loss of localization mechanisms in inflammation and coagulation due to infection, thus interrupting a vicious circle [18,19,20]. For the earliest possible diagnosis of developing sepsis, which is considered a prerequisite for targeted therapeutic intervention, a combination of clinical observations and findings with various biomarkers has been recommended [21]. The path from infection, which at the beginning, in many cases, is not clearly identifiable, to sepsis is via organ dysfunction, which can occur at various sites and is the result of microcirculatory disturbances, most of which can be assessed and monitored including the hematologic system [22,23]. Preclinical studies have shown that in the so-called thrombo-inflammation of sepsis, among others, microcirculatory dysfunction is an early phenomenon [24], in which the endothelial glycocalyx plays a key role [25].

Endothelial glycocalyx acts as a mechano-transducer, mediating shear stress-dependent responses in endothelial cells, including the release of nitric oxide and prostacyclin, which are endothelial-derived inhibitors of platelets. Measurement of circulating and/or urinary glycocalyx components including syndecans, hyaluronan, and heparan sulfate as biomarkers in septic humans has confirmed the degradation of glycocalyx [25]. An association between syndecan-1 levels and DIC in patients with sepsis was reported [26]. The close thrombo-inflammatory relationship between microvascular circulatory dysfunction and coagulation is confirmed by experimental interventions in an ischemia-reperfusion model, showing that antithrombin prevents glycocalyx degradation, sustaining vascular barrier function and preventing edema formation [27].

In clinical medicine, a large proportion of experimental biomarkers are not yet available for routine diagnostics. The current research focus, therefore, is on the simultaneous evaluation of several markers known from sepsis pathophysiology to be significant and measurable in blood [21], as well as their possible combination with direct microcirculation measurements [24].

Table 1 presents an overview of the development of DIC scoring systems over time.

#### 2.1.1. Overt and Non-Overt DIC

Diagnostic criteria for DIC were first defined by the JMHW in 1983 [28] followed by the ISTH criteria for overt and non-overt DIC in 2001 [10]. Whereas the JMHW scoring included a combination of laboratory parameters and clinical features, ISTH scoring relied only on laboratory parameters, including D-dimer, as an alternative to fibrin/fibrinogen degradation products (FDP) and increased the importance of fibrin-related markers but reduced the weight of thrombocytopenia. Both scoring systems identified patients with an increased mortality risk due to DIC.

Non-overt DIC scoring, also proposed by the ISTH in 2001, predicts a high risk of death as well as later development of overt DIC in intensive care unit patients. ISTH non-overt DIC scoring, which incorporates AT levels as a specific marker, predicts death and overt DIC significantly earlier than non-overt DIC scoring without the use of AT data [29]. There is plenty of evidence that decreases in AT levels are likely due to consumption [30], degradation [31], extravasation [32], and reduced production [33], all of which are probably related to DIC, directly and indirectly. Despite early validation of the predictive potential of ISTH non-overt DIC scoring using AT as an additional marker, this scoring system has rarely been used in clinical practice. Replacing the PT difference parameter from the ISTH non-overt DIC score with the specific TAT complex concentration enabled the validation of an improved pregnancy-specific non-overt DIC scoring system more recently [34].

#### 2.1.2. JAAM and Sepsis-Induced Coagulopathy Scorings

The overt DIC criteria were designed to categorize definitive DIC associated with a critical hemostatic disorder and organ dysfunction. However, early detection has been suggested because coagulopathy is recognized as an important cause of organ dysfunction [35]. As preceding coagulopathy contributes to the progression of various diseases, simplified diagnostic criteria to determine the early phase of acute DIC are needed. As a result, the JAAM criteria were specifically designed for acute DIC [36], mainly sepsis-associated DIC, and are widely used in Japan [37]. However, the JAAM criteria have not been adopted by others outside Japan. Instead, the Scientific Standardization Committee of the ISTH released a new category of early-phase DIC arising from sepsis, the sepsis-induced coagulopathy (SIC) scoring system [11]. Criteria that can determine the preceding compensated phase of DIC were integrated in JAAM and SIC, which cover the thrombosis type of DIC but are not suitable for detecting early stages of bleeding (enhanced fibrinolysis)-type DIC, such as in trauma and obstetric emergencies.

### 2.2. Management of Sepsis-Induced Coagulopathy with Endogenous Anticoagulants

The activities of key physiological endogenous anticoagulants, thrombomodulin, and AT decrease in SIC. The decrease in activity is due to a combination of consumption and loss by trans-endothelial escape, among other factors [38]. The stabilization of anticoagulant levels was evaluated.

Different disease-inducing stimuli have been studied in animal models of DIC. However, with comparable coagulation activation, very different patterns of responses have been described for fibrinolytic activity, organ dysfunction, inflammation status, and bleeding symptom, which differed markedly, e.g., between endotoxin- and tissue factor-induced DIC models in rats [39]. Therefore, a careful characterization of the model is required for an experimental and clinical use of such data, which are mentioned here as examples.

Targeting anticoagulation therapy with AT concentrate for the prevention of microthrombus formation, early administration of recombinant AT maintained vascular integrity and the microcirculation by preserving the glycocalyx in an endotoxin-induced rat-model of sepsis, effects that were more prominent with high-dose therapy [40]. A single low dose of recombinant human AT did not prevent DIC occurrence, severity, inflammatory profile, or hemodynamic alterations in a pig model of endotoxin shock [41]. In contrast, in a trauma DIC model in pigs, AT administration was able to attenuate prothrombin complex-induced DIC [42]. The focus of most of these studies has been on biomarkers, and concurrent detection of microvascular clot formation has not been reported; however, given the pathophysiological importance of intravascular thrombus formation in sepsis-associated DIC, such studies should be performed to strengthen the power of these models.

Despite numerous randomized controlled trials (RCTs) on the efficacy of endogenous anticoagulant administration in sepsis-associated DIC with different study designs in humans, the results have remained inconsistent; however, the studies included all patients with sepsis, but not all had concomitant associated DIC. In studies with better designs, improved outcomes were observed in patients with SIC or sepsis-associated DIC treated with anticoagulant therapy [43,44] and as already found in subgroup analyses of large RCTS with evidence for greater mortality reduction in patients with coagulopathy or DIC [45,46,47]. In the SCARLET study on thrombomodulin in sepsis, a coagulopathy phenotype with reduced platelet count and increased international normalized ratio (INR) was defined as an inclusion criterion to demonstrate the efficacy of thrombomodulin administration [3].

Platelets are activated in sepsis; however, treatment attempts with antiplatelet therapy have not been successful [48]. For successful anticoagulant therapy, it will be important to better identify the right patient population with SIC [49].

#### 2.2.1. Antithrombin

**Figure 2:**
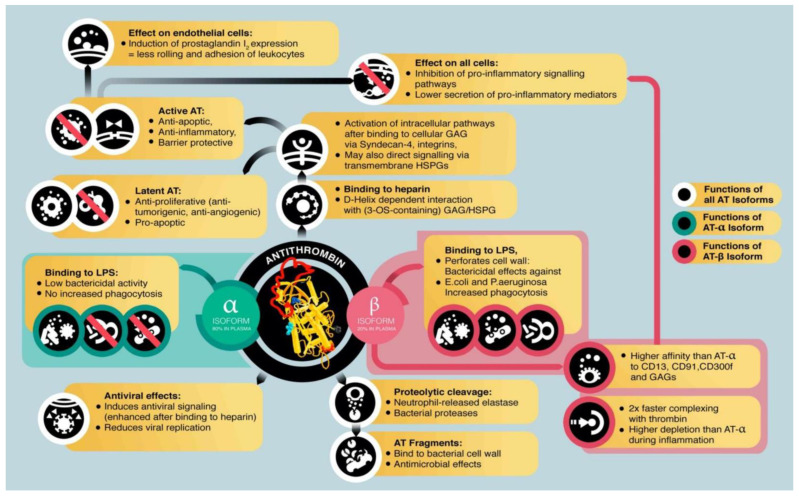
Functions and effects of antithrombin in host defense and inflammation. Note: AT, antithrombin; LPS, lipopolysaccharide; HSPG, heparan sulfate proteoglycans; GAG, glycosaminoglycans. Reproduced without modification from Schlömmer et al. 2021 [50] (accessed on 24 September 2022), under a Creative Commons Attribution-NonCommercial-NoDerivatives 4.0 International (CC BY-NC-ND 4.0) License (https://creativecommons.org/licenses/by-nc-nd/4.0/ (accessed on 24 September 2022). Copyright © 2021, The Authors. This reuse has not been endorsed by the licensor. The source reference is “[50]” and is available at https://www.mdpi.com/1422-0067/22/8/4283.

As a serine protease inhibitor, AT manifests as an anticoagulant, primarily by inhibiting thrombin and activated factor X. AT also has direct inflammation-modulating properties and may produce antimicrobial effects that all provide biological plausibility to the therapeutic effectiveness of AT as an anti-DIC intervention in SIC (Figure 2) [50]. AT interacts with activated proteases of the coagulation system and heparan sulfate proteoglycans on the cell surface. In addition to inhibiting the activated proteases of the coagulation cascade, AT interacts with heparan sulfate proteoglycans on the surface of various cells. As an endogenous anticoagulant serpin, AT is synthesized and released by the liver and mediates the therapeutic effects of administered heparin [34].

AT produces part of its inflammation-regulating properties by directly affecting signal transduction of inflammatory cells, and the inflammatory response of endothelial cells and leukocytes is influenced by the binding of AT to structures of the glycocalyx (syndecan-4), thereby affecting receptor-mediated cell responses [38,51,52,53,54]. In addition, depending on glycosylation, AT can bind to and perforate the cell walls of bacteria, ultimately producing antimicrobial effects [50]. Certain segments of the amino acid sequence of AT, which can be released after proteolytic metabolism, have antimicrobial activity [55].

Recent research has also indicated the antiviral potential of AT. The serine protease TMPRSS2 is important for the virulence of SARS-CoV-2 because it enables the entry of the virus into cells; AT, as a serpin, inhibits this enzyme and could ultimately not only attenuate the viral load in COVID-19 patients, but also SARS-CoV-2 infection at an early stage [56].

In light of the increasing knowledge of the importance of coagulation activation and the role of endogenous anticoagulants in sepsis, the potential beneficial effects of administering AT concentrates as sepsis therapy have been increasingly studied. Clinical trials investigating the efficacy and safety of supraphysiological dosing of AT in sepsis patients have, to this date, failed to confirm a significant mortality reduction [57]. Post hoc analyses from the KyberSept [5] study, which included only sepsis patients with DIC, suggested both improved organ function and lowered mortality when receiving AT compared to placebo (e.g., 28-day mortality 25.4% vs. 40.0%, RR 0.64, 95% CI 0.43 to 0.94, *p* = 0.024) [45]. This has also been supported by retrospective data from Japan [58] and meta-analyses [47]. From this perspective, interventions against DIC based on the favorable influence in addition to the SOFA score of surrogate parameters from coagulopathy including platelet count and PT as well as AT activity or TAT complexes could play a role in the supportive care of patients with sepsis. Its efficacy remains to be confirmed by evidence of mortality reduction in a prospectively controlled manner [59]. To date, supplementation therapy with AT for sepsis has been approved in Japan [19] but is not commonly used elsewhere [20].

#### 2.2.2. Endogenous Anticoagulant Supplementation in Sepsis-Induced Coagulopathy

Recent clinical trials of AT administration in sepsis-associated DIC have shown that with this supplementation, the duration of DIC could be shortened, and survival may be prolonged without the risk of bleeding being increased by the anticoagulant [47]. Given the great prognostic importance of DIC in sepsis, it is important that the promising results of therapeutic trials with endogenous anticoagulants be continued to confirm and optimize their clinical use. In SCARLET and KyberSept, the two largest high-quality RCTs with endogenous anticoagulants at low risk of bias, comparable beneficial results were obtained in sepsis patients with the coagulopathy phenotype, comparable interactions with concomitantly administered exogenous heparin were observed, and dependencies of efficacy evidence on disease severity were observed, confirming that thrombomodulin and AT are of potential importance in the treatment of SIC and sepsis-associated DIC [60]. Using JAAM-defined DIC in sepsis as an inclusion criterion in an RCT showed no effect of AT against death [61] indicating that DIC alone was not sufficient to select optimal targets for anticoagulant therapies.

These results need to be confirmed in further RCTs. In line with the findings of the aforementioned studies, the design of these confirmatory trials with AT and/or thrombomodulin should be based on the definition of SIC, disease severity, and the interaction between endogenous anticoagulants and exogenous heparin.

## 3. COVID-19-Associated Coagulopathy

**Figure 3:**
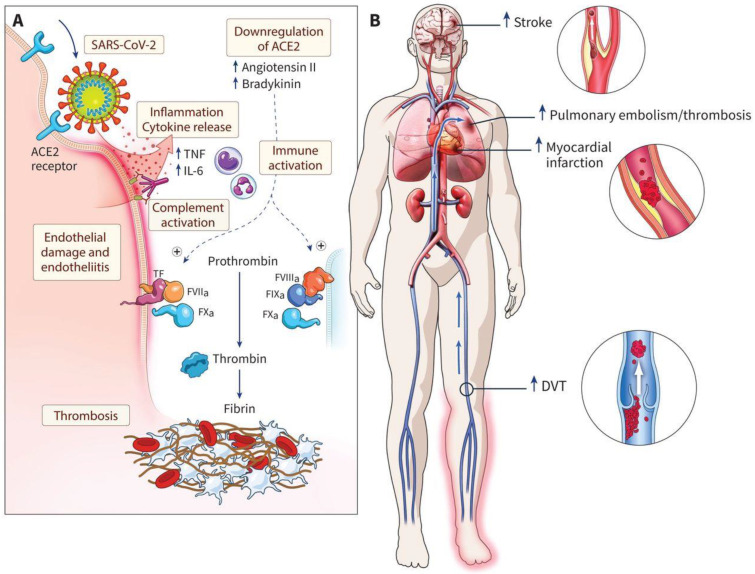
Possible mechanisms for thrombosis in coronavirus disease 2019 (COVID-19) and clinical consequences. (**A**) Injury to the endothelium initiated by severe acute respiratory syndrome coronavirus 2 (SARS-CoV-2) entry into cells via the angiotensin-converting enzyme 2 (ACE2) receptor is thought to lead to diffuse endotheliitis. The endothelial damage may result in an inflammatory host response characterized by excessive immune activation and cytokine storm, which promotes hypercoagulability and thrombosis. (**B**) Possible venous and arterial thrombotic complications associated with COVID-19. Original illustration by freelance medical illustrator Gail Rudakevich. Reprinted with permission from Ref. [15]. Copyright ©2021, Canadian Medical Association. Abbreviations: DVT, deep vein thrombosis; FVIIa, factor VIIA; IL-6, interleukin 6; PE, pulmonary embolism; TF, tissue factor; TNF, tumor necrosis factor α.

SARS-CoV-2 infections cause acute respiratory distress syndrome, which, via local clot activation in the lung, may progress to a thromboembolic disease, which is ultimately not uncommon and is a potentially catastrophic disease manifestation [62]. Biomarker studies support the notion that endothelial dysfunction plays an important role in thromboembolic COVID-19 progression [63]. Compared to other forms of respiratory failure, COVID-19 leads to an increased frequency of venous and arterial thromboses (Figure 3), and prophylactic anticoagulation is broadly recommended [15].

Mechanisms involved in COVID-19-associated coagulopathy revealed novel pathways, including inhibition of thrombomodulin by Angiopoietin-2 [64]. Patients with COVID-19 have altered laboratory values, indicating endothelial damage. If DIC scores are also positive, mortality significantly increases. DIC-like coagulopathy parameters have been reported in COVID-19 in a growing number of studies, in association with increased mortality [65,66]. However, compared with sepsis-associated DIC, the predictive power of ISTH overt DIC scores is reduced in COVID-19 coagulopathy, and D-dimer levels and PT prolongation are more reliable parameters for monitoring disease severity [67].

### Antithrombin in COVID-19-Associated Coagulopathy

In patients who did not survive COVID-19, non-overt DIC was observed by Anwar et al. [67] more frequently (94.8%) than overt DIC (5.2%). Studies on DIC diagnostic criteria and anticoagulant therapy in COVID-19-associated coagulopathy were limited to the ISTH overt DIC or SIC scores and heparinoids, particularly low molecular weight heparin (LMWH) [68]. Although some research is already available, further studies are needed to better understand the prognostic significance of scoring in COVID-19-associated coagulopathy.

Referring to a possibly specific role of AT in COVID-19-associated coagulopathy, TAT complexes are known to increase with D-dimer remaining normal [69]. Testing for AT levels in patients with COVID-19 revealed a significant difference between ICU and non-ICU patients, showing a significant decrease in AT activity in patients with severe COVID-19, 89.65% in all patients, and significantly lower AT activity in the non-survivor group (87.52%) than in the survivor group (92.38%) [70]. Regular testing of AT activity in patients with COVID-19 has therefore been suggested and, if deficient, both a mechanistically alternative non-heparin-dependent form of anticoagulation [71] or AT replacement therapy may be warranted [72]; however, measurement of AT activity in patients with COVID-19 has not been listed among biomarkers of thrombotic risk in a consensus statement from the International COVID-19 Thrombosis Biomarkers Colloquium [16]. Although AT (and protein C) plasma levels may drop, especially in patients who do not survive, they are rarely lower than 80% of the normal levels [65].

Data on AT supplementation therapy for COVID-19-associated coagulopathy are scarce. Fresh frozen plasma (FFP) use in patients with COVID-19-associated coagulopathy showing improved thrombosis prophylaxis with a possible impact on survival [73] may suggest AT-related effects. The efficacy of AT concentrate administration on COVID-19-associated coagulopathy has been investigated in an RCT in adult patients with COVID-19 and an AT activity of <100% as inclusion criteria. This single-center study was terminated prematurely because of insufficient recruitment of the planned 75 patients after the inclusion of 52 patients. Although D-dimer elevation and PT prolongation were numerically reduced in the AT-treated group, these (and other coagulation) endpoints were not significantly altered [74].

Unfractionated heparin (UFH) and LMWH require AT to exert anticoagulation [75], and in AT deficiency, anticoagulation with UFH or LMWH at usual doses may remain inadequate. Case reports of severe COVID-19 highlight that acquired AT deficiency may contribute to both the development of thrombosis and insufficient therapeutic anticoagulation with heparin; therefore, alternative anticoagulation independent of AT may improve outcomes. The measurement of AT and institution of argatroban as alternative anticoagulants that work independently of AT if levels are low may be a suitable alternative for acute anticoagulation in patients resistant to heparin in COVID-19 [76].

## 4. Lessons Learned from COVID-19-Associated for Sepsis-Induced Coagulopathy

In the pre-COVID-19 area, classical DIC presented as thrombo-hemorrhagic diathesis with marked thrombocytopenia, very prolonged PT/APTT, marked elevation of D-dimer, and low fibrinogen as signs of excessive and dysregulated activation of thrombin generation too late for intervention; pre- and non-overt DIC identified as coagulopathy early with decreasing platelet count, prolonging PT/APTT, increasing D-dimer, and decreasing fibrinogen may be the best time for anti-DIC intervention [77].

Several factors characterize the pathophysiology of COVID-19-associated coagulopathy. Behind the high frequency of thrombosis is endothelial inflammation and an increase in the activities of coagulation factors, such as fibrinogen and factor VIII [78]. Arterial thrombosis and venous thromboembolism are more frequent in COVID-19-associated coagulopathy than in SIC or sepsis-associated DIC. The characteristics of bacterial SIC and DIC differ from those of COVID-19-associated viral coagulopathy. COVID-19-associated coagulopathy typically exhibits increased D-dimer and fibrinogen levels, but initial abnormalities in PT and platelet count are minor. An overview of the clinical and laboratory characteristics of COVID-19-associated coagulopathy, SIC, sepsis-associated DIC, hemophagocytic syndrome, antiphospholipid syndrome, and thrombotic microangiopathy is shown in Table 2 [66].

According to several studies, although AT levels are lower in non-survivors of COVID-19 than in survivors, AT activity rarely falls below 80% of the normal levels [65]. However, in ventilated, critically ill patients, AT levels have also been significantly reduced, but there is little evidence to suggest DIC, such as PT, platelet count, and fibrinogen levels [76].

Although COVID-19-associated coagulopathy is different from SIC and sepsis-associated DIC, coagulation test screening including D-dimer and fibrinogen is recommended, as is supportive coagulation management including established thromboembolic prophylaxis and other standard support measures for critically ill ICU patients with SIC or sepsis-associated DIC [62].

## 5. Conclusions

Early detection of coagulopathy is a prerequisite for the successful therapeutic control of the catastrophic consequences of overt DIC. For this purpose, diagnostic algorithms have been repeatedly applied to quantify the status of coagulation activation, platelet count, and fibrinogen conversion. The diversity of triggers and mechanisms leading to DIC in COVID-19 and sepsis needs to be addressed with improved diagnosis of early, non-overt DIC, because until now, the focus has been mainly on overt DIC, which may already have become therapeutically inaccessible. Therapeutic strategies for patients with DIC involve not only resolution of the eliciting triggers, but also supportive therapy for hemostatic imbalance, including heparin, thrombomodulin, and AT. The major reduction in AT levels in SIC rather than COVID-19-associated coagulopathy indicates that AT-dependent mechanisms are likely to play a special role in the progression of SIC to overt DIC. Treatment of SIC with endogenous anticoagulants must be timely and requires diagnostic scoring systems capable of early detection of the initiated progression of SIC to DIC. For this purpose, the ISTH has recently proposed an SIC scoring algorithm. Whether similarly specific criteria as in ISTH non-overt DIC scoring, such as TAT complexes, thrombomodulin, or AT activity, can provide important additional information in personalized patient identification for optional anticoagulant therapy of sepsis must be clarified in prospective studies.

## Figures and Tables

**Table 1:** Japanese Ministry of Health and Welfare DIC, International Society on Thrombosis and Haemostasis Overt DIC and Non-Overt DIC, Japanese Association of Acute Medicine DIC, and Sepsis-Induced Coagulopathy Scoring Systems.

	Points	JMHW	ISTH Overt	ISTH Non-Overt ^+^	JAAM	SIC
Platelet count (×10^9^ × L^−1^)	3	<50	---	---	<80 ≧50% decrease within 24 h	---
	2	≧50, <80	<50	---	---	<100
	1	≧80, <120	≧50, <100	<100	>120, ≦80 ≧50% decrease within 24 h	≧100, <150
	1	---	---	Falling within 24 h	---	---
	–1	---	---	Raising within 24 h	---	---
FDP (mg/dL), D-dimer (mg/dL)	3	>40 FDP ^‡^	Strong increase	---	≧25 D-dimer ^§^	---
	2	>20, ≦40 FDP ^‡^	Moderate increase	---	---	---
	1	>10, ≦20 FDP ^‡^	---	Increased	≧10, <25 D-dimer ^§^	---
	1	---	---	Raising within 24 h	---	---
	–1	---	---	Falling within 24 h	---	---
Prothrombin time	2	>1.67 ratio	≧6s	---	---	>1.4 ratio
	1	>1.25, ≦1.67 ratio	≧3 s, <6 s	≧3 s	≧1.2 ratio	>1.2, ≦1.4 ratio
	1	---	---	Raising within 24 h	---	---
	–1	---	---	Falling within 24 h	---	---
Fibrinogen (mg/dL)	2	<100	---	---	---	---
	1	≧100, <150	<100		---	---
SIRS score	1	---	---	---	>3	---
Total SOFA score ^$^	≧2		---	---		≧2
	1		---	---		1
Symptoms, underlying disease	2	---	---	Disease associated with DIC	---	---
	1	Hemorrhage * or organ dysfunction ^†^	---	---	---	---
	Provided	---	Disease associated with DIC	---	---	---
Specific criteria (antithrombin, protein C, TAT complex)	1	---	---	Abnormal	---	---
	−1	---	---	Normal	---	---
Total score for DIC or SIC		≧7	≧5	≧5	≧4	≧4

^+^ Non-overt DIC suggested by ISTH overt DIC score < 5 (confirmed by ISTH non-overt DIC scoring after 24 h). ^§^ Use convert chart. ^†^ Sepsis-related organ failure (SOFA) score of >2. * Abnormal bleeding independent of the original disease. ^$^ The total SOFA score is the sum of the four items (respiratory, cardiovascular, hepatic, and renal SOFA). The total SIC score was the sum of the SOFA score and coagulation criteria, exceeding 2. ^‡^ If in routine testing laboratories the determination of D-dimer concentrations is performed more frequently than that of fibrin/fibrinogen degradation products (FDPs) and the assays used for D-dimer are different from each other, cut-off values may be given as follows: 0 points, for values up to the upper limit of the standard; 2 points, for values between 1 and 45 times the upper limit of the standard; 3 points, for values exceeding 5 times the upper limit of the standard. DIC, disseminated intravascular coagulation; ISTH, International Society on Thrombosis and Haemostasis [10]; JAAM, Japanese Society on Acute Medicine [36]; JMHW, Japanese Ministry of Health and Welfare [28]; PT, prothrombin time; SIC, sepsis-induced coagulopathy [12]; SIRS, systemic inflammatory response syndrome; SOFA, sequential organ failure assessment; TAT, thrombin-antithrombin.

**Table 2:** The similarities and the differences in thrombosis and laboratory data between COVID-19 and differential diseases.

Coagulopathy	Primary Cause and Target of Coagulopathy	Thrombo-Embolism	Platelet Count	D-Dimer	PT/aPTT	Fibrino-Gen	Anti-Thrombin	Activated Complement System/VWF	Anti-Phospholipid Antibody	Inflammatory Cytokines (IL-1β, IL-6)
COVID-19	Macrophage/endothelial cell	Microthrombosis/venous thrombosis	↑∼↓	↑	→∼↑	↑	→	+	+	↑
DIC/SIC	Macrophage/endothelial cell	Microthrombosis	↓	↑	↑	→∼↓	↓	−	−	↑
HPS	Inflammatory cytokines	Microthrombosis/venous thrombosis	↓	→	→	→	→	−	−	↑
APS	Antiphospho-lipid antibody	Arterial/venous thrombosis	↓	→	PT →	→	→	−	+	−
					aPTT ↑					
TMA (aHUS/TTP)	Complement system/ADAMTS13	Microthrombosis/arterial/venous thrombosis	↓	→∼↑	→	→	→	aHUS +/−TTP −/+	−	−

Arrows indicate direction of change; “+/−” indicates presence or absence. Abbreviations: DIC, disseminated intravascular coagulation; SIC, sepsis-induced coagulopathy; HPS, hemophagocytic syndrome; APS, antiphospholipid syndrome; TMA, thrombotic microangiopathy; aHUS, atypical hemolytic uremic syndrome; TTP, thrombotic thrombocytopenic purpura; PT, prothrombin time; aPTT, activated partial thromboplastin time; VWF, von Willebrand factor; IL, interleukin. Reproduced without modification from Iba et al. 2022 [66] under a Creative Commons Attribution-NonCommercial-NoDerivatives 4.0 International (CC BY-NC-ND 4.0) license (https://creativecommons.org/licenses/by-nc-nd/4.0/ (accessed on 28 September 2022). Copyright © 2020, The Authors. This reuse was not endorsed by licensor. The source reference is [66] and is available at https://ccforum.biomedcentral.com/articles/10.1186/s13054-020-03077-0 (accessed on 28 September 2022).
